# Understanding how animal groups achieve coordinated movement

**DOI:** 10.1242/jeb.129411

**Published:** 2016-10-01

**Authors:** J. E. Herbert-Read

**Affiliations:** 1 Department of Zoology, Stockholm University, SE-10691 Stockholm, Sweden; 2 Department of Mathematics, Uppsala University, S-75106 Uppsala, Sweden

**Keywords:** Collective motion, Collective behaviour, Interaction rules, Leadership, Social responsiveness

## Abstract

Moving animal groups display remarkable feats of coordination. This coordination is largely achieved when individuals adjust their movement in response to their neighbours' movements and positions. Recent advancements in automated tracking technologies, including computer vision and GPS, now allow researchers to gather large amounts of data on the movements and positions of individuals in groups. Furthermore, analytical techniques from fields such as statistical physics now allow us to identify the precise interaction rules used by animals on the move. These interaction rules differ not only between species, but also between individuals in the same group. These differences have wide-ranging implications, affecting how groups make collective decisions and driving the evolution of collective motion. Here, I describe how trajectory data can be used to infer how animals interact in moving groups. I give examples of the similarities and differences in the spatial and directional organisations of animal groups between species, and discuss the rules that animals use to achieve this organisation. I then explore how groups of the same species can exhibit different structures, and ask whether this results from individuals adapting their interaction rules. I then examine how the interaction rules between individuals in the same groups can also differ, and discuss how this can affect ecological and evolutionary processes. Finally, I suggest areas of future research.

## Abstract (precis)

**Summary:** This review highlights the general principles of how animal groups achieve coordinated movement. Differences in the interaction rules of animals within and between species are discussed.

## Introduction

Schools of fish, flocks of birds and marching insects achieve coordination (see Glossary) without choreography. Individuals often coordinate their movements to reduce predation risk through dilution or confusion effects (Krause and Ruxton, 2002). In other cases, collective motion (see Glossary) may be an adaptation to improve foraging success (Bazazi et al., 2012) or to pool information about the direction of new feeding, breeding or nest sites, thereby improving migration efficiency (Codling et al., 2007; Seeley and Buhrman, 1999; Grünbaum, 1998). Collective motion also emerges through antagonistic contacts between individuals, such as when Mormon crickets (*Anabrus simplex*) or juvenile desert locusts (*Schistocerca gregaria*) chase and avoid conspecifics in cannibalistic interactions (Simpson et al., 2006; Bazazi et al., 2008; Hansen et al., 2011). The reasons that other animals move in groups remain unresolved; for example, at high densities, plant–animal worms (*Symsagittifera roscoffensis*) form rotating mills, the function of which remains unclear (Franks et al., 2016). In other systems, collective motion may emerge through repeated interactions between individuals in confined environments (Mann et al., 2013).

The explanations for how animals can achieve these levels of coordination have ranged from mere coincidence to thought transference among individuals (Selous, 1931). We now know that individuals in moving groups respond to the local movements and positions of their neighbours, and these ‘interaction rules’ (see Glossary) between individuals produce coordinated movement. The term ‘interaction rules’ is adopted from earlier work on self-propelled particle models (Box 1). These rules may alternatively be thought of as ‘decisions’, which determine how information gathered from multiple sensory inputs affects an individual's motor response. These inputs could include direct visual or mechanical stimuli from conspecifics, or indirect social cues such as air or water turbulence created by others' movements. Thinking about these decisions as ‘rules’, therefore, can provide a useful framework for understanding how animals in groups achieve coordinated movement.

Glossary **Coordination (in motion)**

The synchronisation of individuals' movements in time and space.

**Collective motion/movement**

When individuals move together in groups through non-independent interactions with one another.

**Directional organisation**

The degree to which individuals align with each other's headings in groups. Neighbours that have low angular deviation in headings are more polarised than neighbours that have high angular deviation in headings. Lower directional organisation is characteristic of swarming states, whereas higher directional organisation in characteristic of milling and flocking states.

**Interaction rules**

How individuals adjust their movements depending on information gathered from their local neighbours' movements and positions. The term ‘interaction’ describes the non-independence between individuals' movements.

**Spatial organisation**

How individuals position themselves with respect to one another in groups. Neighbours can be located uniformly in all directions relative to a focal individual (isotropy) or in particular directions relative to a focal individual (anisotropy). Individuals often attempt to maintain separation distances between one another, resulting in density regulation within groups.

**Tracking**

The process by which the position (and sometimes orientation) of an animal is measured in space and time.

Box 1. A brief history of collective motion The concept of interaction rules can be traced back to the 1950s, when Breder proposed that schooling fish have attraction and repulsion forces that maintain the distance between neighbouring individuals (Breder, 1954). With the development of general-purpose computers in the 1980s, it became feasible to simulate groups of individuals interacting according to such simple rules. In 1982, Aoki simulated particles that adjusted their direction according to the position of their neighbours, moving away from one another at close distances, aligning at intermediate distances and moving towards each other at greater distances (Aoki, 1982). Particles obeying these simple rules formed cohesive and coordinated moving groups, even though individuals were not following specific individuals and did not know all of the other particles' movements or positions. Later, computer game developers wanted to simulate the behaviour of animal groups without having to code individual trajectories. Reynolds (1987) provided a solution, implementing a flocking model with interacting particles. Like Aoki's model, this included terms such as collision avoidance, velocity matching (speed and direction) and attraction, with particles only having local information. Aoki and Reynolds determined that simple movement rules between neighbouring individuals could generate cohesive and coordinated motion, much like that of real animal groups.

Other researchers began to implement variations in the interaction rules that particles obeyed (Huth and Wissel, 1992; Niwa, 1994) and assess the properties of these models (Vicsek et al., 1995). Romey (1996) and Couzin et al. (2002) varied the rules of individuals, giving particles different repulsion and attraction strengths in a group. Couzin et al. (2002) also investigated interaction rules in three dimensions; changing the size of the zones of alignment and attraction resulted in groups displaying different configurations. Other slight variations in the models had interesting effects on the collective patterns that emerged (e.g. altering body shape and the size and shape of interaction zones affects spatial sorting in simulated groups; Kunz and Hemelrijk, 2003; Hemelrijk and Kunz, 2005; Hemelrijk and Hildenbrandt, 2008; Romey and Vidal, 2013). Bode et al. (2010) investigated how group-level properties such as the speed and distribution of neighbours changed when individuals altered how often they updated their position. Other detailed models captured the dynamics and spatial organisation of bird flocks (Hildenbrandt et al., 2010; Cavagna et al., 2015; Lukeman et al., 2010), fish schools (Hemelrijk and Kunz, 2005; Kunz and Hemelrijk, 2003) and insect swarms (Romanczuk and Schimansky-Geier, 2012). For reviews, see Vicsek and Zafeiris (2012) and Sumpter (2010).

How individuals interact in moving groups ultimately affects their survival and reproductive success. Hence, deciphering these interactions allows us to understand the selective pressures that have shaped these social behaviours. These social interactions are also likely to affect how individuals within and between groups mix in populations (Morales et al., 2010), potentially affecting larger evolutionary processes such as speciation. At a finer level, the high temporal and spatial resolution data collected from moving animal groups allows us to map individuals' behavioural responses to their sensory inputs. Combining detailed movement analysis with modern techniques to monitor neural activity (Grover et al., 2016) will produce key insights into the neural mechanisms that govern seemingly complex social behaviours. Furthermore, we may also understand the genetic basis underlying social behavioural differences by mapping detailed behavioural variation in how individuals interact in groups to the associated genetic variation between individuals. This level of understanding, linking genetics, neuroscience and behaviour, can only be achieved if we can accurately measure how individuals interact within groups.

Owing to the large amounts of trajectory data that can be collected using automated methods, we are now amassing information that describes how animals interact in moving groups. This Review will describe how these trajectory data can be used to infer social interaction rules. It will then describe variation in the interaction rules between species, between groups and between individuals, before finally exploring the implications of this variation, suggesting possible paths for future research.

## Analysing interaction rules in animal groups

Models have been useful for understanding the general principles of collective motion (see Box 1), but they are no substitute for data collected from real animal groups. Methods for collecting data on the positions and movements of individuals in groups have been around for some time (Radakov, 1973; Partridge, 1980, 1981), but automated tracking technologies (see Glossary) based on computer vision (Cavagna et al., 2008a,b; Branson et al., 2009; Straw et al., 2011; Pérez-Escudero et al., 2014; Handegard and Williams, 2008) and miniature GPS (Nagy et al., 2010) have made it easier to collect vast amounts of data from individuals in groups. Furthermore, statistical physics has provided tools to analyse these trajectories and determine how individuals within groups are interacting (Cavagna et al., 2008c; Nagy et al., 2010). These methods now allow the investigation of interaction rules in real animal groups, without relying on predictive computer simulations.

**Fig. 1.**
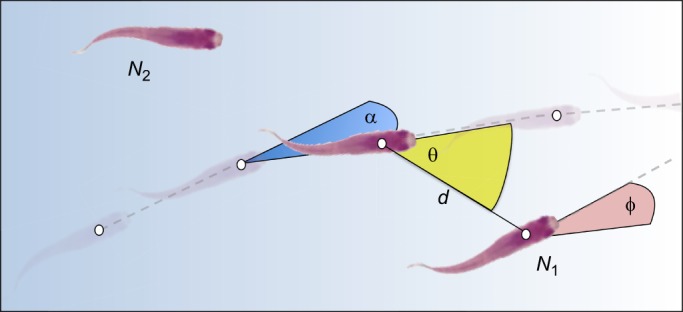
**Tracking data can provide information on the relative positions and orientations of individuals in groups.** From these data, the distance (*d*), direction (θ) and differences in heading (φ) between a focal individual and its neighbours (*N*_1_ or *N*_2_) can be quantified. The acceleration, speed or turning angle (α) of the focal individual can then be correlated with these measurements to infer how individuals in groups are responding to each other's movements and positions.

Tracking tools provide information on the position (usually the centre of mass) of animals within their environment. One of the first patterns to investigate with these data is the relative positions of individuals with respect to one another. When animals move along a horizontal plane, the position of a neighbour (*N*_1_) relative to a focal individual can be described by two measures: the bearing angle (θ) and the distance to that neighbour (*d*; Fig. 1). The proportion of time for which neighbours are located at certain distances and bearings represents the likelihood of finding neighbours at those positions. Because the spatial distribution of individuals with respect to one another is fundamentally linked to the rules that individuals use to interact, these relative positions can be particularly informative for inferring not only which individuals are interacting, but also how they are interacting.

If positional readings are taken over time, then the movements of an animal between two points can be inferred. The vector between two successive positions gives information on that animal's speed and direction. By factoring in the movements and positions of other individuals in the group, the movements of individuals with respect to one another can be measured (Fig. 1). This reveals how individuals are adjusting their position and orientation in response to their neighbours' movements and positions. This is done, for example, by correlating an animal's acceleration or turning angle with the position, orientation or other movements of its neighbour (Lukeman et al., 2010; Katz et al., 2011; Herbert-Read et al., 2011; Pettit et al., 2013). These fine-scale movement responses can be scaled to the whole group; individuals' directional changes can be used to infer how quickly information is propagated across groups (Attanasi et al., 2014a; Herbert-Read et al., 2015a). Changes in the speed and direction of individuals over time can also highlight other dynamics occurring within moving groups. For example, leader–follower relationships can be inferred by seeing whether the speed or direction changes of a focal individual are adopted (after some time delay) by neighbouring individuals. If individual A's direction (or speed) is copied by conspecific B, then we can infer that B was following A. Thus, we can build a picture of which individuals are more influential in guiding group movement (Nagy et al., 2010). Fine-scale tracking data, therefore, allow us to infer the rules that individuals are using to maintain cohesion and order in moving animal groups.

It is important to note that these rules are proxies for how an animal perceives information about its surroundings and integrates this information to then perform a motor response. Nevertheless, correlations between certain stimuli (e.g. distance to neighbour) and output (e.g. change in speed) reveal basic but important principles governing an individual's decision to move. It will be important to find more biologically meaningful sensory inputs that govern these behavioural responses, for example, by correlating the retinal size of an object or neighbour with an animal's velocity (Boeddeker et al., 2003). Indeed, newer analytical techniques that consider the body shape and potential visual fields of individuals in groups will lead to a deeper understanding of the sensory inputs that elicit behavioural movement responses (Strandburg-Peshkin et al., 2013; Rosenthal et al., 2015). Nevertheless, basic measures such as the distance to and speed of a neighbour are inextricably linked to the sensory cues that individuals use to detect the movement and positions of neighbours. They can, therefore, reveal important insights into the similarities and differences between how individuals interact in moving animal groups.

## Differences and similarities between species

### Spatial organisation

**Fig. 2.**
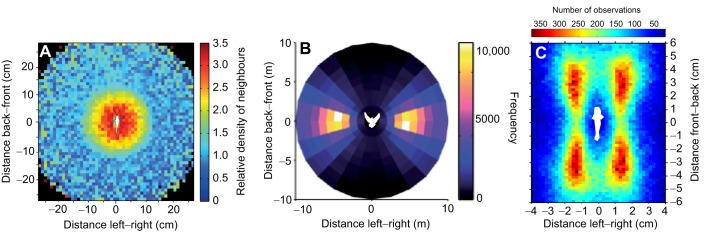
**The relative density of neighbours around a focal individual for different species.** The focal individual is represented by the white silhouette in the centre of each plot (not to scale) and is facing in its direction of motion. (A) Locusts are commonly found 3–10 cm apart and in any direction surrounding a focal individual. Lower densities of neighbours occur within a 1 cm region close to an individual. Adapted from Buhl et al. (2012). (B) Pigeons (*Columba livia*) are commonly found side by side with respect to their direction of motion. Adapted from Pettit et al. (2013). (C) Female guppies (*Poecilia reticulata*) in pairs position themselves in front of or behind, and to the left or right of a partner (author's unpublished data).

One of the striking differences between animal groups is the spatial organisation (see Glossary) of individuals within them. For locust nymphs that move along the ground, the distribution of nearest neighbours around a focal individual is uniformly distributed in all directions, with neighbours most commonly 3–10 cm apart (Fig. 2A) (Buhl et al., 2012). This type of distribution is referred to as spatially isotropic. In other species that move along a horizontal plane, nearest neighbours are more often located in specific directions relative to a focal individual (spatially anisotropic). Surf scoters (*Melanitta perspicillata*), which swim across the water's surface, have clearly defined nearest-neighbour distributions. Birds seldom come within 1 body length of one another and are most commonly found 1.45 body lengths apart, in front of or behind one another (Lukeman et al., 2010). When the positions of pairs of fish relative to one another are measured in the horizontal plane, mosquitofish (*Gambusia holbrooki*), golden shiners (*Notemigonus crysoleucas*) and minnows (*Phoxinus phoxinus*) position themselves in front of or behind their neighbour at ∼1.5–2 body lengths apart (Herbert-Read et al., 2011; Katz et al., 2011; Partridge, 1980). Other species, such as giant danios (*Danio aequipinnatus*), homing pigeons (*Columba livia*) and starlings (*Sturnus vulgaris*), position themselves more often side by side with respect to their direction of motion (Fig. 2B) (Grünbaum et al., 2005; Pettit et al., 2013; Ballerini et al., 2008). Pairs of female guppies (*Poecilia reticulata*) most often position themselves in diagonally offset configurations (Fig. 2C). In some species, however, the distribution of nearest neighbours often becomes more isotropic as group size or density increases (Partridge, 1980; Partridge et al., 1983; Katz et al., 2011).

For animals that can also move in the vertical plane, this additional direction of motion allows individuals to vary their relative elevations. Although pairs of minnows position themselves one in front of another with little difference in their elevation, as school size increases, individuals increase the distance between near neighbours in the vertical plane (Partridge, 1980). In effect, individuals position themselves slightly above or below one another. Similar results have been found in saithe (*Pollachius virens*), herring (*Clupea harengus*), dunlin (*Calidris alpina*), starlings (*Sturnus vulgaris*) and mysid shrimp (*Tenagomysis oculata*) (Partridge et al., 1980; Major and Dill, 1978; Ballerini et al., 2008; O'Brien, 1989). Because of the difficulties of collecting trajectory data in three dimensions, it is currently unclear how individuals integrate information about their neighbours' position and movements in the vertical plane – this will be briefly returned to later on.

The spatial positions individuals adopt in groups may be partly determined by the available sensory modalities (Box 2) or the energetics associated with moving in groups (Box 3). In many cases, however, whether there are functional benefits for individuals adopting specific positional preferences remains unclear (Hemelrijk and Hildenbrandt, 2012). Nevertheless, the relative positions that individuals adopt in groups can be used to infer which individuals are interacting. Ballerini et al. (2008) measured the degree of anisotropy between successive nearest neighbours in starling flocks. They found that the relative positions of the eighth and further nearest neighbours did not differ from random. This occurred regardless of a flock's density. This is consistent with individuals interacting with a fixed number of near neighbours (termed topological interactions) instead of reacting to individuals within some distance of each other (metric interactions). The dichotomy between topological and metric interactions, however, may not be absolute; individuals may move away from a specific individual that is too close, but may be attracted towards multiple individuals further away. In a more sensory-based approach, Strandburg-Peshkin et al. (2013) examined golden shiner schools, and found that the likelihood of an individual responding to the movements of others was best predicted by assessing which neighbours could see each other. By assessing the visual network of these fish, Rosenthal et al. (2015) went on to predict the magnitude of information propagation across these schools. Therefore, assessing the basic structure and positions that individuals adopt in animal groups can reveal which individuals are interacting in groups, and highlights that the range and type of these interactions, perhaps unsurprisingly, differs between species.

Box 2. Sensory determinants of spatial organisation Differences in the spatial organisation of groups may be partly linked to the sensory modalities animals use to interact. *Temnothorax* ants (*Temnothorax albipennis*) perform tandem runs, where one ant leads another to a new nest site or food source (Pratt, 2008; Franks et al., 2002). Franklin et al. (2011) found that vision was not necessary to coordinate this movement, whereas tactile interactions and pheromones were. Fitzgerald and Pescador-Rubio (2002) also found that tactile stimuli and pheromones were important in establishing and maintaining single-file processions in social caterpillars (*Hylesia lineata*). The separation distances between whirligig beetles (*Dineutes discolor*) are controlled by both antennal detection of surface waves produced by nearby conspecifics and visual cues (Romey et al., 2014). Similarly, the separation distances between locusts are controlled by visual and tactile interactions (Bazazi et al., 2008). Because locusts have 360 deg vision in the horizontal direction (Rogers et al., 2010), they may use this information to maintain minimal separation distances between neighbours, but without directional preferences for where those neighbours are (Buhl et al., 2012). In contrast, animals that position themselves at specific bearing angles with respect to one another may have limitations to where neighbours can be detected (Hemelrijk and Hildenbrandt, 2012). For example, starlings have a minimal blind angle behind the head of 32 deg (Martin, 1986), and may attempt to position neighbours where they can see them (Ballerini et al., 2008). However, this does not explain why some species of fish, which also have blind angles, position themselves more often in front or behind one another (Herbert-Read et al., 2011; Katz et al., 2011). Instead, individuals may attempt to position neighbours in regions of their field of view where changes to the optic flow, looming rates or other movements of neighbours can be more easily detected (Dill et al., 1997). Other specialised organs, such as the lateral line in some species of fish, allow individuals to maintain separation distances (Pitcher et al., 1976). If the lateral line organ is cut, saithe (*Pollachius virens*) tend to position themselves side by side, rather than in their regular front–behind configuration (Partridge and Pitcher, 1980). Many species, therefore, integrate multiple sensory inputs to determine neighbours' locations, and which sensory inputs are available can affect the movements and spatial positioning of individuals in groups.

Box 3. Energetic determinants of spatial organisation The hydrodynamic or aerodynamic effects associated with moving in groups are sometimes important in determining the spatial positions that individuals adopt (Killen et al., 2011; Marras et al., 2015). For example, model simulations predict that individuals in fish schools swim more efficiently in ‘diamond’ configurations (Weihs, 1975; Hemelrijk et al., 2015a). In nature, bald ibises (*Geronticus eremita*) occupy positions in V formations that are consistent with aerodynamically optimal positions (Portugal et al., 2014). Pelicans (*Pelecanus onocrotalus*) have reduced heart rates and wing-beat frequencies when flying in V formations than when alone (Weimerskirch et al., 2001). Therefore, in some groups, individuals may occupy positions that reduce their energy expenditure when on the move. However, when the density of individuals changes, but their directional preference does not, the hydrodynamic or aerodynamic benefits of grouping cannot explain such positioning behaviour (Ballerini et al., 2008). Indeed, for pigeons, there are energetic costs associated with flying in flocks, suggesting other reasons for why pigeons flock (Usherwood et al., 2011).

**Fig. 3.**
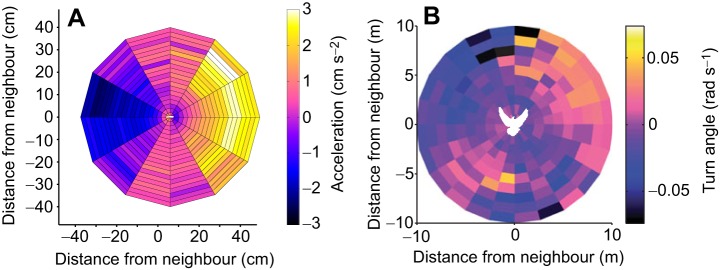
**The acceleration and turning responses of mosquitofish (*Gambusia holbrooki*) and pigeons (*Columba livia*).** (A) Acceleration response of a fish in the centre of the plot and facing right, as a function of distance from its neighbour. There is a region of repulsion close to the focal individual, and attraction occurs at distances further away. Together, these act to maintain distances between individuals within the group. Adapted from Herbert-Read et al. (2011). (B) Pigeons turn towards or away from their neighbour depending on the position and distance of that neighbour. Positive turning angles represent turns to the right, whereas negative turning angles represent turns to the left. Pigeons will turn towards their neighbour when it is >3 m away, and away from their neighbour when it is <3 m away. Adapted from Pettit et al. (2013). The fish and bird silhouettes in the centre of the plots are not to scale.

The spatial positions individuals adopt in groups allows us to infer not only who is interacting with whom, but also how these individuals are interacting. If individuals have preferred positions that they wish to maintain, then movements to correct deviations from these positions can be thought of as interaction rules. Perna et al. (2014) investigated how the interaction rules of simulated groups would appear if individuals had different spatial preferences with respect to their neighbours. If animals preferred to maintain front–back configurations with some separation distance, individuals would accelerate towards neighbours far ahead of them, and decelerate when neighbours were directly in front of them (Perna et al., 2014). Indeed, animals that maintain front–back configurations adopt these predicted movement rules. The interaction rules of golden shiners and mosquitofish have been analysed in detail (Katz et al., 2011; Herbert-Read et al., 2011). Pairs of fish mainly position themselves in front of and behind one another. Fish are rarely observed within 1 body length of one another, and are most often ∼1.5–2 body lengths apart. The fish modulate their speed depending on their neighbour's position. When their neighbour is directly in front of them (<1.5 body lengths), they decelerate, and when their neighbour is directly behind them (<1.5 body lengths), they accelerate. When their neighbour is >1.5 body lengths in front of or behind them, they accelerate or decelerate, respectively. The fish have close to zero acceleration when their neighbour is ∼1.5 body lengths in front of or behind them (Fig. 3A). These changes in speed, based on neighbours' positions, have also been observed in juvenile blacksmith (*Chromis punctipinnis*) and zebrafish (*Danio rerio*) (Parrish and Turchin, 1997; Zienkiewicz et al., 2015).

**Fig. 4.**
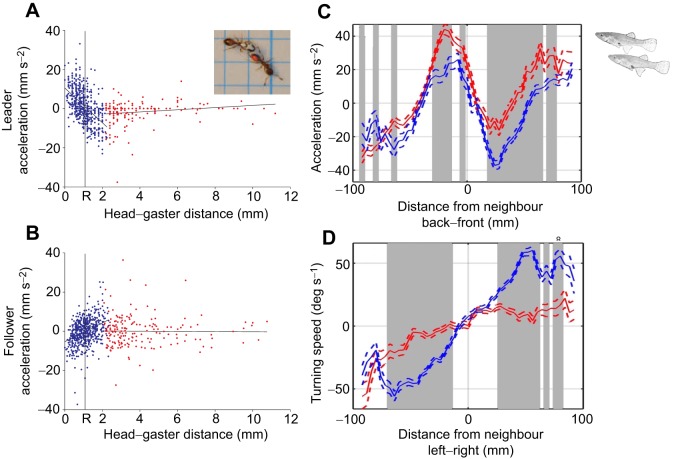
**Leader and followership in pairs of ants (*Temnothorax alibipennis*) and mosquitofish (*Gambusia holbrooki*)**. (A,B) Acceleration responses of the leader (A) or follower (B) ant, depending on the distance to their partner. When the pair are <∼1 mm apart (R=1 mm), the leader will accelerate, whilst the follower will decelerate (blue circles left of R). When the pair are between ∼1 and 2 mm apart, the follower will accelerate and the leader will decelerate (blue circles right of R). Both leader and follower show close to zero acceleration when >2 mm apart (red circles). Adapted by permission from Macmillan Publishers Ltd, Nature; Franks and Richardson (2006). (C) Mean change in speed and (D) mean change in angle of mosquitofish that were leaders (solid red curve) or followers (solid blue curve) as a function of their partner's location. In C, negative *x* regions indicate that the focal individual (either leader or follower) was behind its partner and positive *x* regions indicate that the focal individual was in front of its partner. In D, negative *y* regions indicate that the focal individual was to the left of its partner, and positive *y* regions indicate that it was to the right of its partner. Dashed curves are plotted one standard error above and below all means in each panel. These responses show how individuals differentially adjust their velocity as a function of their neighbours' location. Grey regions represent the regions where the movement responses of leaders and followers are significantly different. Adapted from Schaerf et al. (2016).

Acceleration and deceleration responses are not only important in maintaining separation distances between fishes, but also crucial in maintaining coordination during tandem running in *Temnothorax* ants (Franks and Richardson, 2006)*.* During these runs, the follower attempts to maintain antennal contact with the lead ant. When the leader's gaster (or tip of her abdomen) is within twice the length of the follower's antennae, the follower decelerates, whereas the leader accelerates. However, when the ants are more than this distance apart, the leader decelerates and the follower accelerates (Fig. 4A,B). These simple behavioural rules, which can be thought of as repulsion and attraction forces, allow the pair to move whilst remaining together. In many species, changes in speed are particularly important for maintaining order and cohesion when individuals move together.

Changes in speed are not the only way for an individual to adjust its position relative to its neighbours. Individuals can also adjust their direction. Indeed, in addition to changes in speed, golden shiners and zebrafish adjust their direction depending on the position of their neighbours (Katz et al., 2011; Zienkiewicz et al., 2015). An individual will turn away from a neighbour that is positioned <1 body length and directly to the side of itself, acting to maintain a minimum distance between pairs. Lukeman et al. (2010) also found that the headings of birds in front of or behind one another deviated strongly in surf scoter flocks. This could be attributed to individuals avoiding neighbours that were directly in front of them. Army ants (*Eciton burchelli*) also use turning responses along their foraging trails to avoid oncoming neighbours (Couzin and Franks, 2003). If an ant returning along the trail collides with an outgoing ant, the outgoing ant will turn away from the incoming ant, giving the returner priority in its direction of travel. This asymmetric interaction rule can produce lanes of traffic, minimising congestion along the trail. Turning responses can be used, therefore, to move away from neighbours that are too close in order to maintain separation distances.

When neighbours are far away from one another, however, turning responses can also act to bring individuals back together. When golden shiners or zebrafish are >1 body length apart, they turn towards their neighbours (Katz et al., 2011; Zienkiewicz et al., 2015). The turning responses of mosquitofish are similarly dependent on the position of their neighbours. Fish turn left when their neighbour is on their left, and turn right when their neighbour is on their right (Herbert-Read et al., 2011). In another study on barred flagtails (*Kuhlia mugil*), fish kept relatively smooth trajectories and adjusted their turning angle as a function of their time to collision with the wall and their neighbour's position (distance and angle towards neighbour) (Gautrais et al., 2012). Pettit et al. (2013) also found that pigeons changed their angle of heading depending on the distance to their neighbour. A pigeon will turn towards a neighbour >3 m away, and will turn away from a neighbour <3 m away (Fig. 3B). As well as speed changes, turning responses depending on neighbours' positions maintain the spatial organisation of individuals in bird flocks, fish schools and along ant trails. Which rules animals adopt is likely to depend on how easy it is to change speed or direction in the medium the organisms are travelling through, whilst at the same time maintaining control.

### Directional organisation

Above, I discussed how individuals can maintain their relative positions in groups by changing their speed and direction depending on their neighbour's position. However, groups show not only spatial organisation, but also directional organisation (see Glossary). That is, individuals in groups often move in the same direction, with small angular deviations in their heading. This could occur if individuals changed their direction to align with that of their neighbours. To detect alignment responses, an individual's turning response (α in Fig. 1) should be correlated with the direction its neighbour is facing (Φ in Fig. 1), and not only with that neighbour's position (θ and *d* in Fig. 1). In some species, these effects can be observed. Barred flagtails, for example, tend to align with their neighbours' direction of travel (Gautrais et al., 2012). Pigeons similarly take into account the direction in which a neighbour is heading when deciding in which direction to turn (Pettit et al., 2013). If a neighbouring pigeon is to the immediate left of a focal individual and is turning right, then the focal individual is more inclined to turn right as well. In effect, partners close to one another match their orientations. Perna et al. (2014) predicted that animals that exhibited alignment responses would often position themselves side by side, and indeed this is observed for pigeons (Fig. 2B).

Directional organisation is also observed when honeybee swarms are guided to a new nest site (Beekman et al., 2006). Schultz et al. (2008) and Latty et al. (2009) concluded that uninformed swarm members are attracted towards fast-flying ‘streaker’ bees that provide directional information on the new nest site's location. However, it is unclear whether bees move towards or align with these fast-flying neighbours. Other species, such as golden shiners and mosquitofish, form polarised groups even though individuals do not appear to explicitly align with their neighbour's direction of travel (Katz et al., 2011; Herbert-Read et al., 2011). Individuals of both species turn towards the location of their neighbour (when the fish are not trying to move apart), but not towards the direction this neighbour is facing. Buhl et al. (2012) also found that pairs of locusts <13.5 cm apart travelled in the same direction; however, whether individuals explicitly aligned with their neighbours was equivocal. Earlier models of locust marching and fish schooling often included alignment terms (Buhl et al., 2006; Yates et al., 2009; Couzin et al., 2002), but lack of evidence for explicit directional matching has inspired a new set of models that do not include these terms (Romanczuk and Schimansky-Geier, 2012; Strömbom, 2011). These models demonstrate that groups can exhibit directional organisation even without explicit alignment terms. Instead, directional organisation is brought about through a combination of repulsion and preferential attraction to others in lead positions (Katz et al., 2011).

## Group and individual variation

Although there are similarities and differences in the structure of groups and their underlying interactions between species, some of the most striking differences in group structure and organisation are observed between groups of the same species. Below, I will discuss how different group states can exist with identical interaction rules. I will then explain how and why individuals might adjust the interaction rules they use in groups, and highlight the consequences this has for a group's structure. This will first assume that individuals within a group are interacting using identical rules. Second, I will explore whether individuals within the same group differ in how they interact with one another, and discuss the implications this has for the ecology and evolution of moving groups.

### Group variation

Animal groups can undergo rapid transformations from milling states, with individuals circling round a central core, to polarised states, with individuals travelling in the same direction at the same speed, to swarming states, with low directional alignment between neighbouring individuals. Golden shiners, for example, transition between swarming, milling and polarised states; however, certain states are more common for different group sizes (Tunstrøm et al., 2013). Smaller groups (30 individuals) are more often found in polarised states, whereas larger groups (300 individuals) more often adopt milling states. It may be tempting to think this occurs because individuals change how they interact in different group sizes. In fact, simulations show that these two states can exist with exactly the same interaction rules (Couzin et al., 2002); indeed, there is no evidence that golden shiners change how they interact in differently sized groups (Katz et al., 2011). Multiple stable collective states, therefore, can emerge from identical interaction rules.

Although the specific rules that individuals use to interact may not change with group size, the frequency of interactions can (Sumpter et al., 2008). If space is limited, increasing group size will naturally increase the group's density. Individuals in larger schools of squid (*Illex illecebrosus*), for example, are closer together than in smaller schools (Mather and O'Dor, 1984). This will increase the frequency of interactions between individuals because of the increased likelihood of collisions. For locusts, the nature of interactions does not appear to change with group size (Buhl et al., 2006). Instead, more frequent interactions between locusts at higher densities result in larger (and denser) groups being more ordered (Buhl et al., 2006). Similar findings are reported for tadpoles (*Xenopus laevis*), where denser groups are also more ordered than sparser groups (Katz et al., 1981). The velocities of individual midges (*Dasyhelea flavifrons*, *Corynoneura scutellata* and *Cladotanytarsus atridorsum*) are more correlated when they are closer together ([Bibr JEB129411C3][Bibr JEB129411C4]), although this is probably not due to individuals attempting to avoid one another, but rather the sexual interactions that occur within these swarms (Okubo and Chiang, 1974). The density of individuals in groups, therefore, can affect the frequency of interactions between individuals and thereby the group's directional organisation.

In addition to changes in the frequency of interactions, the strength of these interactions can also change. Here, the strength of interactions can be interpreted as the likelihood of individuals responding to or copying the movements or headings of near neighbours. These changes may be thought of as adaptions to individuals' interaction rules. The strength of interactions is often linked to the speed at which individuals are travelling in groups. Fishes, for example, will attempt to match their speed with that of their neighbours (Partridge, 1981), as this presumably reduces the likelihood of collisions. At higher speeds, individuals weight the orientational information of neighbours more strongly (Gautrais et al., 2012), which is consistent with the observation that faster groups are often more polarised (Tunstrøm et al., 2013; Viscido et al., 2004). It is important to note here, however, that groups can also transition from unpolarised to polarised states even if they remain stationary (Partridge, 1980). This highlights that individuals can change how strongly they weight the directional information of neighbours even without changes to their speed, in turn affecting the directional organisation of groups.

Increases in speed are often a result of some external perturbation, such as a predator's attack. Rapid accelerations following an attack are observed in insects (Treherne and Foster, 1981), crustaceans (O'Brien, 1989), fish (Herbert-Read et al., 2015a) and birds (Beauchamp, 2012). Because increases in speed often make groups more polarised, in turn, polarised states may be more effective at transmitting information between group members. This occurs because individuals can quickly copy their neighbours' deviations from current velocities (Bialek et al., 2014), resulting in ‘waves’ of information travelling through groups (Procaccini et al., 2011; Hemelrijk et al., 2015b). Alternatively, individuals may adapt their speed according to their nutritional state, and this can affect large-scale group dynamics. Bazazi et al. (2011) found that although an individual locust's movement was largely unaffected by its nutritional state when isolated, when in groups, locusts fed low-protein diets moved ∼40% faster than locusts fed a high-protein diet. These changes in speed could be explained by individuals changing the strength of their social interactions. Because desert locusts cannibalise conspecifics, it was hypothesised that individuals attempting to pursue and escape one another did so more strongly when deprived of protein. By simulating particles with different interaction strengths, Bazazi et al*.* (2011) found that those with stronger social interactions (protein-deprived individuals) formed more-ordered groups at lower densities than weakly interacting particles (protein-satiated individuals). Later, Bazazi et al. (2012) found that spadefoot toad (*Spea multiplicata*) tadpoles were more likely to form a vortex group structure, with tadpoles rotating in a circular movement pattern, when they had been food deprived compared with when they were satiated. These rotating vortexes may be an adaptive response to disturb the substrate, thereby creating new feeding patches for individuals within the group. Different group structures can therefore arise from slight differences in the strength of social interactions, which may result from external perturbations or different nutritional states between individuals.

Individuals can also change the strength of their interactions across their development. For example, some fish form loose aggregations with limited collective movement immediately after hatching, compared with polarised groups during their adult lives (Shaw, 1960). Groups of larger tadpoles of the clawed frog (*Xenopus laevis*) are more polarised than groups of smaller tadpoles (Katz et al., 1981). Adult shrimp (*Paramesopodopsis rufa*) tend to form denser and more polarised schools than juveniles (O'Brien, 1989), and larger squid (*Loligo opalescens*) form more polarised schools than smaller squid (Hurley, 1978). Coordinated behaviour can only emerge when the sensory and motor architecture of individuals are sufficiently developed to allow coordination between neighbours. The development of rods in the eyes of the striped jack (*Pseudocaranx dentex*), for example, coincides with the development of attraction towards conspecifics (Masuda and Tsukamoto, 1996). At this developmental stage, however, individuals do not form polarised groups even though they have developed the manoeuvrability required to school. The transition between merely aggregative behaviour to schooling behaviour, therefore, is unlikely to only be determined by the sensory and motor capabilities of developing individuals (Masuda and Tsukamoto, 1998). How individuals process and respond to the position and orientational information of neighbours also seems to be important. Romenskyy et al. (2015 preprint) asked whether the strength of interactions was different between different-sized fish (*Pseudomugil signifer*). Groups of small fish (∼7.5 mm) formed loose aggregations with lower polarisation compared with groups of medium-sized (∼13 mm) or larger (∼23 mm) fish, which formed highly polarised, compact groups. Thus, although the strength of repulsion was similar for different-sized fish, the attraction strength between individuals was stronger for larger individuals. There is also a suggestion that ibis (*Eudocimus albus*) individuals learn to coordinate their movements with others, with birds gradually flying more often in V formations as they age (Petit and Bildstein, 1986; Biro et al., 2016). The interaction strengths of individuals may change, therefore, as they grow, learn or age.

### Individual variation

Above, I considered that groups show different structures and organisation, and this is linked to the interaction rules of individuals. However, this discussion assumed that all individuals were interacting using identical rules. In fact, individuals within the same group can differ in how they are interacting with one another. The best evidence for this comes from the observation that the spatial sorting of individuals within groups is non-random, with particular individuals occupying consistent positions within groups (Pitcher et al., 1982; Partridge, 1981; Krause, 1994; Freeman et al., 2011). Spatial sorting is important to consider, because individuals that occupy positions at the front of moving groups can often influence group movement more than others (Herbert-Read et al., 2011; Katz et al., 2011; Nagy et al., 2010). In these cases, being at the front of the group is synonymous with leading because directional changes often propagate from front to rear positions. Some of the best examples of leader–follower dynamics are observed in homing pigeon flocks, with individuals located at the front of these flocks having a disproportionate influence on group direction (Nagy et al., 2010, 2013). However, the individuals that emerge as leaders in these flocks are not necessarily the best navigators (Flack et al., 2012; Watts et al., 2016) or more socially dominant (Nagy et al., 2013). Instead, faster, larger birds emerge as leaders (Pettit et al., 2015, 2013; Herbert-Read, 2015). Similar results have been found in other taxa – larger roach (*Rutilus rutilus*), for example, occupy positions at the front of groups (Krause et al., 1998; Krause et al., 2000). Therefore, differences in individuals' body size and self-assortment as a result of preferred speed can lead to certain individuals disproportionally influencing others' movements.

In other cases, it is clear that individuals differ in their interaction rules, as the spatial organisation of groups is not related to differences in body size. Partridge (1981) found that the spatial sorting in shoals of saithe (*Pollachius virens*) was not dependent on body size, but individuals did have preferred positions within groups. Burns et al. (2012) also found that mosquitofish occupied consistent positions within groups, and this was not related to their sex, body size or dominance. In these cases, differences in how individuals interact with their neighbours can drive this spatial sorting. For example, we have already seen that during tandem running in *Temnothorax* ants, leaders and followers use different interaction rules to maintain their position in the pair (Fig. 4A,B) (Franks and Richardson, 2006). Schaerf et al. (2016 preprint) also found that pairs of fish differed in how they interacted with one another. When individuals were close together, ‘leaders’ (individuals most often found at the front of the pair) had higher accelerations when their partner was directly behind them, and lower decelerations when their partner was directly in front of them (Fig. 4C). Thus, the repulsion strength of individuals can be asymmetrical, with some individuals having stronger repulsion in front of than behind themselves. Followers (individuals most often found at the back of the pair) also had higher turning speeds towards their partner's position than leaders (Fig. 4D), indicating that followers were more responsive to their partner's position. Leadership roles have also been observed in other species of fish (Nakayama et al., 2012a,b). Together, these differences highlight that individuals can differ in their likelihood of following others, producing differences in spatial sorting (Couzin et al., 2005) and leadership in a process termed ‘leadership though social indifference’ (Conradt et al., 2009).

Why might some individuals be less responsive to their neighbour's movements and thereby occupy positions at the front of groups? These differences could be state dependent and driven by motivation to feed, as non-satiated fish move faster (Hansen et al., 2015a) and have larger inter-individual distances than their satiated counterparts (Hansen et al., 2015b), and often occupy positions at the front of groups (Krause et al., 1998). McClure et al. (2011) similarly found that hungrier caterpillars were more likely than recently fed conspecifics to be found at the front of groups. Alternatively, the likelihood of following another's movements may relate to intrinsic differences in behavioural phenotype, in part driven by differences in stress physiology or metabolism (Koolhaas et al., 1999; Careau et al., 2008). The bolder of two sticklebacks (*Gasterosteus aculeatus*), for example, is less likely to follow their partner's movements and is more likely to lead the pair (Harcourt et al., 2009; Jolles et al., 2015). We shall now see that these differences can have wide-ranging implications for the ecology and evolution of collective motion.

## Implications of having varying interaction rules in groups

Given that natural selection acts on the variation in individuals' behaviours and phenotypes, could selection act on differences in the way individuals interact in groups, and could this shape group structure and motion? This was first confirmed in computer simulations, when Wood and Ackland (2007) found that fast-moving polarised groups or slow-moving milling groups evolved when particles with different interaction rules were exposed to simulated predation pressure. However, predatory tactics are key for understanding which prey are targeted in groups (Morrell et al., 2015); thus, it was important to determine whether the choices of real predators could select for coordinated movement. Ioannou et al. (2012) achieved this by projecting videos of simulated prey onto the side of an aquarium containing a real predator – a bluegill sunfish (*Lepomis macrochirus*). Prey in the videos interacted with their neighbours differently. Individuals with low attraction towards neighbours and low alignment formed small groups with low tortuosity. In contrast, individuals with intermediate levels of attraction and alignment formed cohesive and coordinated groups. The sunfish preferentially targeted prey in smaller groups, and groups that were less polarised, showing that predation can indeed select for prey with particular interaction rules, in turn selecting for coordinated group movement.

Varying interaction rules not only shape the general properties of moving groups, but can also shape how groups make decisions. Couzin et al. (2005) investigated whether a minority of informed individuals that balanced social interactions with a desired direction of travel could lead a majority of uninformed individuals that only used social interactions. Only a small number of informed group members were required to guide a majority of uninformed individuals. In effect, individuals that relied less on social interactions could guide more socially responsive individuals; predictions from this model were later confirmed in studies of pigeons (Biro et al., 2006), fish (Ward et al., 2008; Couzin et al., 2011) and baboons (Strandburg-Peshkin et al., 2015). Later, Ioannou et al. (2015) asked what made informed individuals effective leaders. They showed that fish with faster, straighter and less variable paths were less effective at guiding groups compared with fish with slower, more tortuous paths. Effective leaders, therefore, need to balance their social interaction strength with their own goal-orientated behaviour. Indeed, individuals still have to be followed if they are to lead (King, 2010). Therefore, by changing their social interaction strengths, individuals can have a disproportionate influence on group decisions.

If individuals can lead others by reducing their social interaction strength, and travel in their own direction of preference without sacrificing group membership, why don't all individuals attempt to do this? In an evolutionary framework, Guttal and Couzin (2010) proposed that there could be costs associated with ignoring social interactions and relying on private information. For example, private information could be costly to acquire because of the energetic investments needed to detect environmental cues, and individuals may neglect important information that is gathered through social interactions, e.g. predator vigilance (Guttal and Couzin, 2011). Their model demonstrated that if these costs were sufficiently high, then populations evolved that consisted of some individuals being highly socially responsive, whilst other individuals adopted a strategy of private information acquisition, with reduced reliance on social interactions (Guttal and Couzin, 2010). Wolf and McNamara (2013) later outlined that frequency-dependent selection could also maintain the numbers of different responsive types in populations. If all individuals in groups completely ignore social information, then they cannot achieve coordination and grouping benefits are lost. Conversely, if all individuals are extremely socially responsive, ignoring their own private information, coordination is achieved, but individuals cannot exploit novel information or resources. Hence, natural selection should favour both socially responsive and unresponsive individuals in populations depending on their relative frequency (Wolf et al., 2008). Whereas leaders (socially unresponsive) gain by imposing their preferences on followers (socially responsive), followers gain by only having to sample social information, and not potentially costly private information (Webster and Laland, 2008). The proportion of leaders and followers in populations should then be determined by the potential for conflict among group members (Johnstone and Manica, 2011). Environments that promote conflict within groups should favour the evolution of socially unresponsive individuals, whereas when conflict is limited or absent, socially responsive individuals should be selected for. Thus, frequency-dependent selection, depending on environmental conditions, can lead to and maintain different levels of social responsiveness in populations (Johnstone and Manica, 2011). These models explain why it is often only some individuals that exert their influence in group decisions, and hint at the evolutionary strategies that may surround differences in individuals' interaction rules.

The social responsiveness of individuals is also likely to be a plastic, context-dependent trait. This flexibility, however, may differ between individuals, and may be reinforced or reversed over time. For example, leaders in homing pigeon flocks learn more about their environment and become better navigators than followers (Pettit et al., 2015). This may reduce leaders' reliance on using social information, exaggerating their leadership role. In other cases, these roles can be reversed, but this depends on which roles are adopted. Nakayama et al. (2013) found that leaders readily adopted follower roles in pairs of sticklebacks, but the adoption of leadership roles by followers was less flexible. Both the flexibility and likelihood of following others, therefore, can vary between individuals. This flexibility may also change over the lifetime of individuals. For example, 1-year-old whooping cranes (*Grus americana*) have a 34% reduction in the distance they travel during migrations if they fly with older birds (Mueller et al., 2013). Although not directly tested, this suggests that younger birds are more responsive to the movements of older birds, and this responsiveness changes as the birds learn their migration route. Differences in the flexibility of interaction rules, and in particular, the likelihood of copying the movements of others, can therefore affect processes such as learning and leadership, and may be intricately linked to the evolution of different socially responsive types in populations.

## Future directions

The field of collective motion has benefited from a surge in acquisition and analysis of data from real animal groups. We should now attempt to integrate this knowledge with both proximate and ultimate explanations of collective motion. At the proximate end of the spectrum, new analytical techniques will allow us to better understand the physiology and genetic basis governing differences in individuals' interaction rules. For example, novel behavioural assays, including animal–robot interactions, can provide identical social conditions to different individuals in different trials (Faria et al., 2010). This will allow us to detect precise differences in how different individuals interact under standardised conditions (Wark et al., 2011). Artificial selection could then be used to select for individuals with particular social interactions, for example, by scoring individuals for their degree of social responsiveness (Szorkovszky et al., 2016 preprint). This would allow us to measure the genetic heritability of these differences (Wark et al., 2011), and by crossing individuals from different populations, the quantitative trait loci associated with different aspects of group behaviour could be identified (Greenwood et al., 2013, 2015; Kowalko et al., 2013). One crucial aspect of this would be to measure differences in levels of hormones, and the expression of genes responsible for hormone production, that are involved in social behaviour. Some key candidates that could regulate social responsiveness are oxytocin, vasopressin and their non-mammalian homologues. These hormones are involved in regulating social behaviour and social information use in animals (Insel and Young, 2000; Donaldson and Young, 2008; Reddon et al., 2014), and may provide a proximate link to differences between individuals' interaction rules. Integrating detailed behavioural responses with genetic, neurological and physiological measures is now key, not only to understanding how individuals interact in moving groups, but also in other areas of evolutionary and behavioural biology (Hofmann et al., 2014).

We should endeavour to understand how animals integrate information on the movements and positions of neighbours in three dimensions. This will require a better understanding of the sensory inputs received from conspecifics (Strandburg-Peshkin et al., 2013; Rosenthal et al., 2015). Tracking systems can now monitor the 3D positions and body orientations of animals in real time (Straw et al., 2011), giving us the opportunity to immerse animals in virtual-reality environments (Fry et al., 2008) with virtual conspecifics. These systems will allow us to control the sensory inputs received from virtual neighbours, revealing how animals perceive and respond to each other's movements in groups. In these systems, we should also endeavour to detect the precise instances when an animal decides to update its position on the move. Approaches to date have usually determined the interaction rules of individuals between every frame at which their positions were recorded; however, in many systems, animals are more likely to make discrete, intermittent decisions to move (Kramer and McLaughlin, 2001). Identifying these decisions will lead to a deeper understanding of the neurological and perceptual processes that underlie group movement.

These data should then be used to inform more data-driven models of collective motion (Calovi et al., 2014), which may require a different approach to the models that have been previously proposed. For example, the field may benefit from building models in a perceptual control-theory framework, instead of a rule-based approach. This framework explains how complex behaviour (such as group movement) can be generated when individuals attempt to stabilise different aspects of their perception, instead of responding to conspecifics using certain rules (Bell and Pellis, 2011). We should also seek new analytical techniques to assess the effectiveness of model fitting (Mann et al., 2014, 2013; Herbert-Read et al., 2015b). These models are important, because they inspire us to build more reliable robotic systems (Sahin, 2004) and develop new algorithms to solve complex collective motion problems, such as how to effectively herd groups of agents (Strömbom et al., 2014).

Much of the data collected on moving groups to date have been gathered under laboratory conditions. However, advances in miniature GPS and sonar will now also allow us to gather high-resolution temporal and spatial data from animals in their natural habitats. Combining movement data with knowledge of habitat structure, resource sites and environmental conditions will allow us to examine the importance of social and environmental cues that allow animal groups to navigate through their environment. This will lead to a deeper understanding of the movement ecology of animal groups.

In addition to the proximate causes of differences between individuals' interaction rules, the ultimate consequences of these differences should be assessed. One fundamental question is how these interaction rules evolved in natural populations. Indeed, the similarities in interaction rules between distantly related species of fishes suggests that some rules governing collective motion have been conserved over time, or that there are constraints for how individuals can interact in groups. Comparing the interaction rules of different species in a phylogenetic framework could prove useful for understanding the evolution and maintenance of particular interaction rules. This could also be investigated by comparing differences in the interaction rules between populations of the same species that have been exposed to different selection pressure over their evolutionary history. Different populations of Trinidadian guppies, for example, have been subject to varying degrees of predation pressure (Magurran, 2005). Fish from high-predation environments form more cohesive groups than fish from low-predation environments (Seghers, 1974). The interaction rules underlying these differences have yet to be determined, and this natural experiment may provide important insights into how predation can select for different interaction rules in groups.

We should ask how variation in the interaction rules of individuals can be maintained in populations, informed and inspired by the predictions of evolutionary models (Wolf and McNamara, 2013; Johnstone and Manica, 2011; Guttal and Couzin, 2010). This will require testing the range of social interactions within and between individuals in natural populations. Importantly, this will need to consider the costs and benefits of different social interactions in different social and environmental contexts. Here, we might benefit from thinking about the interaction rules of individuals in a strategic framework (Laland, 2004). For example, we could consider different interaction rules as strategies that attempt to maximise individuals' survival and reproductive success in different contexts. A functional approach to understanding which interaction rules are adopted in groups would greatly improve our understanding of a field that has, to date, been mechanistically focused. Nevertheless, the analytical techniques outlined here now allow these ideas to be tested.

## Conclusions

In this Review, I have highlighted how animal groups can achieve coordinated motion, and discussed how the interaction rules within and between species, groups and individuals differ. I have also discussed the larger-scale evolutionary and ecological considerations surrounding these differences. With constant improvements in tracking methods and analytical techniques, this area of research will continue to provide considerable insight into other fields. Because we can now measure detailed movement and behavioural responses of individuals in groups, we can combine this knowledge with fields including physiology, neuroscience and genetics to give a more comprehensive understanding of individuals' social behaviour. This integration is unprecedented, and will no doubt lead to exciting breakthroughs in years to come.
